# Targeting c-MYC with T-Cells

**DOI:** 10.1371/journal.pone.0077375

**Published:** 2013-10-10

**Authors:** Florian Helm, Thomas Kammertoens, Frank M. Lehmann, Andrea Wilke, Heiko Bruns, Josef Mautner, Georg W. Bornkamm, Armin Gerbitz

**Affiliations:** 1 Department of Immunology, Charité Berlin, Berlin, Germany; 2 Institute of Clinical Molecular Biology and Tumor Genetics, Helmholtz Center, Munich, Munich, Germany; 3 Department of Pediatrics, Technical University (TU) Munich and Clinical Cooperation Group Pediatric Tumor Immunology, TU Munich and Helmholtz Center, Munich, Germany; 4 Department of Hematology, Oncology, University of Erlangen, Erlangen, Germany; University of Illinois at Chicago, United States of America

## Abstract

Over-expression of the proto-oncogene c-MYC is frequently observed in a variety of tumors and is a hallmark of Burkitt´s lymphoma. The fact that many tumors are oncogene-addicted to c-MYC, renders c-MYC a powerful target for anti-tumor therapy. Using a xenogenic vaccination strategy by immunizing C57BL/6 mice with human c-MYC protein or non-homologous peptides, we show that the human c-MYC protein, despite its high homology between mouse and man, contains several immunogenic epitopes presented in the context of murine H2^b^ haplotype. We identified an MHC class II-restricted CD4^+^ T-cell epitope and therein an MHC class I-restricted CD8^+^ T-cell epitope (SSPQGSPEPL) that, after prime/boost immunization, protected up to 25% of mice against a lethal lymphoma challenge. Lymphoma-rejecting animals contained MHC multimer-binding CD8^+^ cell within the peripheral blood and displayed *in vivo* cytolytic activity with specificity for SSPQGSPEPL. Taken together these data suggest that oncogenic c-MYC can be targeted with specific T-cells.

## Introduction

Cancer driving oncogenes frequently contain mutations in their coding sequences, but in many cases also remain wild-type and acquire their oncogenic property through uncontrolled expression. Since immunogenic mutations within the protein sequence are rare and may differ from patient to patient, T-cell based immunotherapy strategies focus on targeting tumor-associated or self-antigens. Targeting unmutated oncogenes *in vivo* is difficult due to central tolerance. However, by utilizing cross-species barriers in xenogenic immunization approaches, even highly conserved proteins can become immunogenic and stimulate the non-tolerant repertoire of the host, thereby allowing for the identification of T-cell receptors (TCR) with specificity for the oncogenic target [1]. 

The proto-oncogene *c-MYC* plays a crucial role in the pathogenesis of a large number of human tumors including B-cell lymphomas and leukemias as well as a variety of different epithelial tumors [2]. Unlike many other proto-oncogenes whose activity is dependent on mutations, truncation or gene fusion, the oncogenicity of c-MYC is in most cases the result of loss of transcriptional control leading to over-expression and accumulation of the unmutated protein itself. However, mutations within the c-MYC protein, although not a prerequisite for rendering c-MYC oncogenic, have also been observed in a fraction of human B-cell lymphomas [3-5]. In human Burkitt’s lymphoma, mouse plasmocytoma, and rat immunocytoma, activation of the *c-MYC* gene is brought about by chromosomal translocation of *c-MYC* into one of the three immunoglobulin heavy or light chain loci [6]. Thereby, the physiological regulation of the *c-MYC* gene is disrupted and the transcriptional regulatory elements of the immunoglobulin genes gain control over the juxtaposed *c-MYC* gene and govern its expression. In a variety of human epithelial tumors and also a subset of large diffuse B-cell lymphomas, the *c-MYC* gene is over-expressed as a consequence of gene amplification which correlates with poor prognosis [7,8]. Oncogenic activation of c-MYC can also occur through events upstream of c-MYC leading to uncontrolled c-MYC expression as observed for example in familial adenomatous polyposis and in K-RAS induced pulmonary carcinoma [9-11].. It thus appears that many, if not all, routes to cancer converge on c-MYC. 

In several experimental systems, downregulation of c-MYC expression resulted in sustained tumor regression [12-15]. As already indicated, tumors appear to be addicted to c-MYC even if the oncogenic signal is upstream of c-MYC rendering c-MYC an excellent target for cancer therapy [11]. c-MYC is also expressed in proliferating normal tissues like e.g. regenerating gut epithelium and hematopoietic cells. The expectation of severe adverse side effects has therefore hampered the development of therapeutic strategies targeting c-MYC for many years. This view has, however, been challenged recently by several groups [2,16,17] who argued that potential benefits may outweigh the risks of targeting c-MYC. The main two arguments in favor of an anti-c-MYC therapy are that (i) tumors are usually addicted to c-MYC and that even short-term interruption of c-MYC expression may drive tumor cells into apoptosis, rendering sustained anti-c-MYC therapy unnecessary [13], and (ii) that most normal cells are quiescent and side effects of c-MYC inhibiting proliferation of normal cells in the skin, the intestine and the hematopoietic system are relatively weak and reversible, and may be well tolerated [11]. 

T-cells have been proven to be effective for the treatment of a variety of malignant diseases. However, choosing unmutated c-MYC as a T-cell target bears two major obstacles: first, T-cells specific for c-MYC may be present only at low affinity and frequency or may be even non-existent due to negative selection in the thymus; secondly, induction of c-MYC specific T-cells may cause autoimmunity to highly proliferative tissues such as the hematopoietic system or the enteral mucosa. 

Specific eradication of c-MYC over-expressing cells should result in long-term growth arrest and, ideally, in eradication of all malignant cells. To circumvent central tolerance, we aimed to generate T-cells against unmutated human c-MYC protein in C57BL/6 mice. Xenogeneic vaccination has been shown to break tolerance to self, and tumor associated antigens in some experimental models by inducing cross-reactive T cells [1,18,19]. Because murine c-MYC exhibits only 89.9% amino acid sequence identity to its human counterpart, differences in the amino acid sequence may give rise to T-cell responses and leave the function of tissues expressing murine c-Myc unaffected. Until now, human c-MYC has been used an as oncogene in a number of mouse models for induction of different tumors and has, so far, been considered as not immunogenic in the H2b genetic background. Using a murine Burkitt´s lymphoma model over-expressing human c-MYC [20], we show here that immunization of mice with human c-MYC protein and c-MYC derived non-homologous peptides elicits a c-MYC-specific CD4^+^ and CD8^+^ T-cell response. Furthermore, human c-MYC-reactive animals are protected from lethal doses of B-cell lymphoma cells over-expressing human c-MYC.

## Methods

### Mice

C57BL/6 mice were obtained from Charles River Laboratories at 6 weeks of age and housed in single ventilated IVC cages with a maximum of 5 mice per cage. Animals were sacrificed by CO_2_ asphyxiation according to the guidelines of the local administration LaGeSo (Government of Berlin). Immunization and lymphoma transfer experiments were conducted under ethical approval (55.2-1-54-2531-8-04 and 209.1/211-2531-8/04, Government of Bavaria, Munich, Germany) 

### Proteins and Peptides

Peptides (purity 70-95%) were purchased from Thermo Scientific biopolymers (Ulm, Germany). Chicken ovalbumin was purchased from Worthington (Lakewood, USA). To obtain c-MYC protein for vaccination, the human *c-MYC* gene, derived from the translocated allele of the BL60 lymphoma cell line, was amplified by PCR and cloned into the pTrcHis vector (Invitrogen) to generate pTrcMYCHis. *Escherichia coli* DH5α were transformed with pTrcMYCHis, selected clones were expanded and protein expression was induced by isopropyl-β-d-thiogalactopyranoside (IPTG) at a concentration of 1 mM. c-MYC protein was purified from bacterial lysates by Ni-NTA agarose (Qiagen, Germany) according to standard protocols. Protein concentration was determined by micro Lowry (Sigma) and presence of c-MYC protein was confirmed by Western blot using a c-MYC-specific antibody (clone 9E10, Invitrogen).

### Epitope prediction

Epitope prediction for MHC class I-restricted peptides was performed using the HLA restrictor software [21]. Epitopes were chosen depending on their peptide-MHC affinity (<500 nM) and the presence of at least one difference in the amino acid sequence between mouse and man. For MHC class II epitopes, the NetMHCII algorithm was used [22]. 

### Immunization of mice

Mice were vaccinated with 50 µg protein (human c-MYC or OVA) or alternatively c-MYC-derived peptides (90 µg), together with CpG ODN1826 (50 µg) (TIBMolBiol) in 50 µl PBS and 50 µl incompletes Freund´s adjuvant (Sigma-Aldrich). The vaccine was injected into the flanks adjacent to the inguinal lymph nodes. 

### *In vivo* depletion of CD25^high^ cells

Monoclonal anti-CD25 antibody (clone PC61, kind gift from Elisabeth Kremmer, Helmholtz-Center Munich) was injected i.p. at a dose of 10 µg/g body weight [23]. Blood was taken 6 days past injection to confirm depletion of regulatory T-cells by staining of CD25 and FoxP3-positive CD4^+^ cells. Mice were immunized 7 days after depletion with PC61-antibody and boosted three weeks after prime immunization.

### *In vivo* cytotoxicity assay

*In vivo* cytotoxicity assays were performed as described previously [24]. Splenic target cells from C57BL/6 mice were isolated and either loaded with target peptide (10 µM) for 15 min at 37°C or used as unloaded controls. CSFE-labeling was performed for 15 min at 37°C at a concentration of 0.1 µM CSFE (high) for peptide-loaded target and 0.01 µM CSFE (low) for unloaded control cells. Both cell populations were mixed at a 1:1 ratio and 2x10^7^ cells were injected *i.v.* in a volume of 200 µl PBS. Peripheral blood was obtained 18 h after injection.

### IFNγ ELISPOT assay and IFNγ ELISA

ELISPOT plates (Millipore) were coated for 12 hours using IFNγ capture antibody (clone AN18, Mabtech) at a concentration of 15 µg/ml in PBS. After 12 hours, 1x10^4^ APC per well were seeded in RPMI1640 supplemented with 10% FCS. Full length OVA or c-MYC protein was added at a concentration of 10 µg/ml and incubated for 24 hours at 37°C. 1x10^5^ MACS-purified (Miltenyi) CD90.2 (Miltenyi, Germany) purified T-cells from c-MYC-immunized mice were added and cultured for 48 hours. Spots were visualized by IFNγ detection antibody (clone R4-6A2) and streptavidin-linked alkaline phosphatase (3321-2A, Mabtech, Germany). The number of spots was calculated using ELISPOT reader ELR02 with respective software (AID, Germany). 

IFNγ concentrations were determined using an IFNγ ELISA (Becton-Dickinson, Germany) according to the instructions of the manufacturer.

### Lymphoma challenge of mice

For tumor challenge experiments, 1x10^5^ 291PC cells [25] were injected subcutaneously into the abdominal flanks. Tumor growth was monitored 3 times per week using a sliding caliper. Mice were sacrificed when tumors reached a diameter of >10 mm. Lymphoma growth curves are plotted as a mean of all animals of one group. 

### Flow cytometric analysis

Cells were washed and incubated for 10 min in ice-cold PBS containing 0.5 µg/ml mAb clone 2.4G2 to block Fc-γ receptors. Fluorochrome-labeled monoclonal primary antibodies were incubated for 30 min at 4°C in the dark. Cells were washed twice in ice-cold PBS and subsequently analyzed (FACSCalibur, Becton Dickinson).

For intracellular staining, cells were cultured for 12 hours in the presence of brefeldin-A. After staining of surface markers, cells were fixed and permeabilized using BD FixPerm (Becton Dickinson) according to the guidelines of the manufacturer. IFNγ was stained for 30 min at 4°C using XMG1.2 antibody. Cells were washed and analyzed subsequently. MHC-multimer staining was performed according to the manufacturer’s instructions (Proimmune). 

### Generation of APC, MCA205MYC-tet cells and coculture with T-cells

APC were prepared as described previously [26]. Briefly, bone marrow cells were prepared from femurs of C57BL/6 mice and kept in Petri dishes for 2 hours at 37°C in RPMI1640 media supplemented with 10% FCS. Non-adherent cells were collected and seeded in six-well plates in complete RPMI medium containing 20% of supernatant from murine GM-CSF secreting NIH3T3 cells [27]. The final GM-CSF concentration ranged between 10-20 ng/ml (GM-CSF ELISA, Becton-Dickinson). For APC-T-cell co-cultures, APC were harvested at day 12 and seeded in 96-well plates at a concentration of 1 to 2.5x10^4^ cells per well. c-MYC, OVA, or peptides were added at a concentration of 2 µg per 100 µl for 12 h. T-cells (1x10^5^) or splenic cells (5x10^5^) were added and incubated with APC for 48 hours at 37°C. To increase uptake and cross-presentation of exogenously added protein [28], the c-MYC-specific mAb 9E10 (kindly provided by Wolfgang Uckert, MDC Berlin) was added in some experiments at a concentration of 0.1 µg/ml.

MCA205 cells [29] were transfected with pBC266 [30], using FuGene transfectant reagent (Promega) according to the manufacturer´s instructions. Cells were treated with hygromycin and one clone was selected to generate MCA205MYC-tet cell line 5x10^4^ cells were used for *in vitro* restimulation of 2.5x10^5^ splenic cells in a 96 well for 48 hours. To downregulate c-MYC expression, tetracycline (1µg/ml) was added 24 hours before coculture.

### Real time PCR

Total RNA was isolated from lymphoma samples using RNeasy Plus (Qiagen) according to the manufacturer's instructions. After DNAse treatment, cDNA was prepared by reverse transcription with random decamers (Applied Biosystems). cDNA was analyzed by quantitative real time PCR (ABI step one) using the following primers (Metabion): 18S RNA forward 5-CGCCGCTAGAGGTGAAATTC-3, reverse 5-CGAACCTCCGACTTTCGTTCT-3. c-myc RNA forward 5-CGCAAGACTCCAGCGCCTTCTC-3, reverse 5-GGCGCTGCGTAGTTGTGCTGATG-3. C_t_ values were quantified using appropriate software (Applied Biosystems) and expressed in arbitrary units (A.U.).

## Results

### Immunization with full-length human c-MYC protein induces IFNγ secreting T-cells

**Figure 1:**
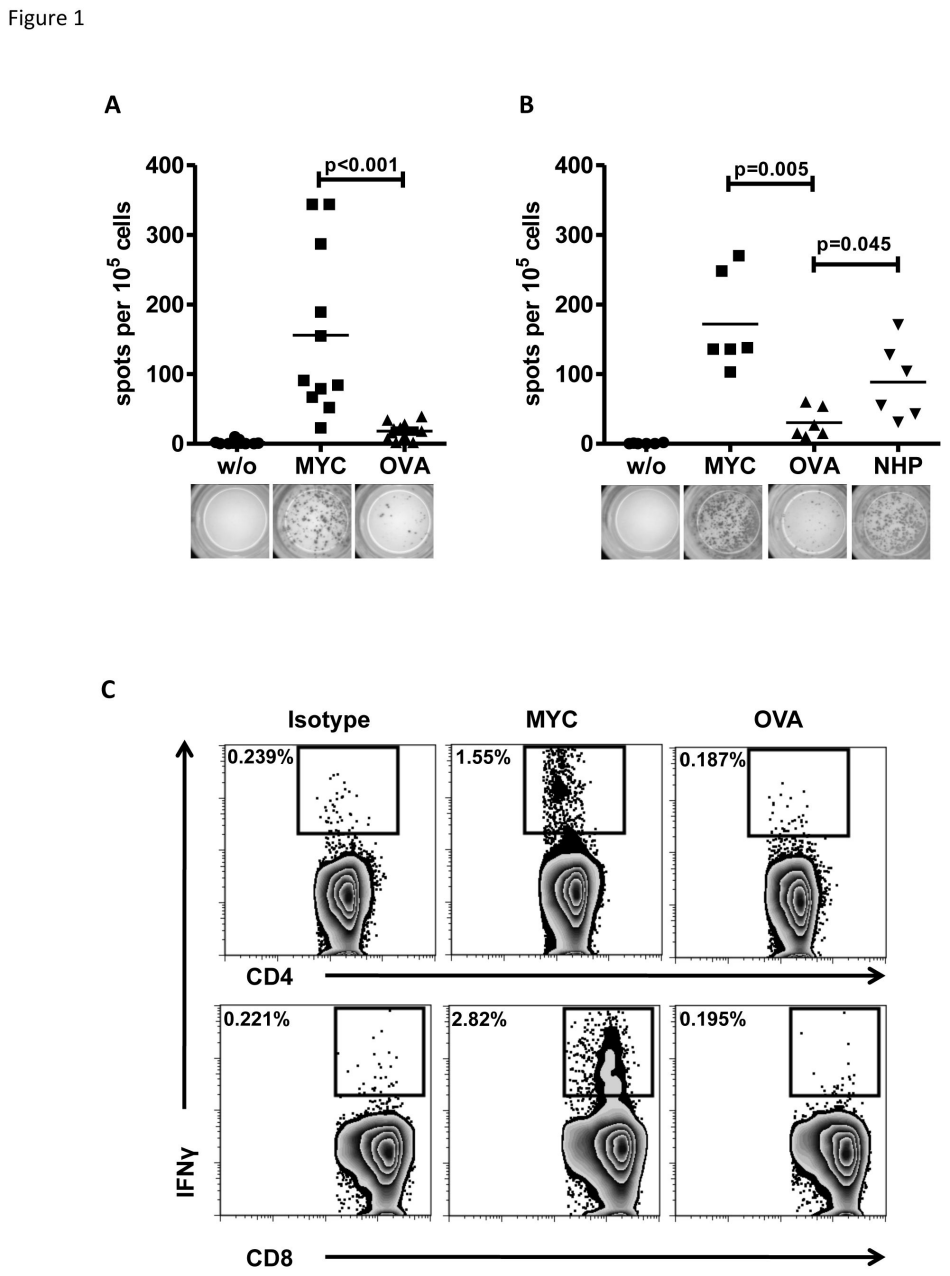
Immunization with human c-MYC protein induces a T-cell response in C57BL/6 mice. A: ELISPOT assay: 1x10^5^ CD90.2-purified T-cells from c-MYC-immunized mice were cultured for 48 hours alone (w/o) or in the presence of 1x10^4^ APC loaded with either c-MYC or OVA protein. Every symbol depicts an individual mouse. Data are compiled from 3 independent experiments (n=11). Representative pictures of ELISPOT wells are depicted below the diagram. B: Purified T-cells from mice immunized with full-length c-MYC protein and boosted with a NHP pool were cultured for 48 hours alone (w/o) or in the presence of 1x10^4^ APC loaded either with c-MYC, OVA or NHP pool. Data are summarized from 2 independent experiments (n=6). C: 5x10^5^ spleen cells from two c-MYC protein-immunized mice were pooled and cultured for 12 hours in the presence of 5x10^4^ protein-pulsed APC. OVA protein served as control. Intracellular cytokine staining for IFNγ revealed presence of MYC-specific T-cells in both, the CD4^+^ and the CD8^+^ T-cell compartment (representative samples from one out of two independent experiments).

The human c-MYC protein (p67) is 89.9% homologous to its murine counterpart. Differences in the amino acid sequence between both proteins are shown in Figure 1. To investigate if differences in amino acid sequences result in immunogenic epitopes in the murine MHC H2^b^ genetic background, we immunized C57BL/6 mice using a vaccine comprised of a full-length c-MYC protein as immunogen and CpG ODN1826 and IFA as adjuvants. Mice were immunized, boosted three weeks later and sacrificed on day 7 after the boost immunization. T-cells from spleen and inguinal lymph nodes of immunized mice were enriched using anti-CD90.2 immuno-magnetic bead separation and stimulated with *in vitro* generated antigen presenting cells (APC) pulsed with either c-MYC protein or chicken ovalbumin (OVA) as unspecific control. The frequency of IFNγ secreting T-cells was determined by IFNγ ELISPOT assay (Figure 1A). Number of spots were significantly higher when T-cells from c-MYC immunized mice were restimulated with c-MYC protein-pulsed APC compared to OVA-pulsed APC (MYC vs. OVA, p<0.001). T-cells cultured without (w/o) APC did not show any spontaneous IFNγ secretion. These results demonstrated that the human c-MYC protein harbors immunogenic epitopes that activate T-cells in the context of murine H2^b^. As a first step to identify T-cell epitopes, peptides were designed based on binding predictions to mouse MHC class I H2K^b^, H2D^b^ or MHC class II H2I-A^b^ (using HLA-Restrictor epitope prediction software [21] (Figure S1). As shown in Table 1, non-homologous peptides (NHP- A-H) contained potential binders to different H2 class I and class II alleles of C57BL/6 mice. To identify specific T-cell epitopes, a peptide pool (NHP A-H) was used for heterotypic immunization in combination with the full-length c-MYC protein. Animals were primed with c-MYC protein and boosted with the peptide pool. On day 28 after first immunization splenic T-cells were CD90.2 enriched and restimulated with either c-MYC-pulsed or peptide pool-pulsed APC. As shown in Figure 1B, the ELISPOT assay for IFNγ revealed that restimulation with peptide pool-pulsed APC resulted in a higher number of spots than restimulation with control protein OVA (p=0.045). When compared to c-MYC-pulsed APC, only 3 out of 6 animals reached a comparable number of spots. However, analysis of supernatants from restimulation cultures by IFNγ ELISA displayed comparable concentrations of IFNγ between cells restimulated with c-MYC protein or peptide pool, whereas T-cells from naïve or OVA-immunized control mice revealed only background secretion levels (Figure S2). From these results we concluded that the non-homologous peptide pool contained the majority of the T-cell reactivity observed previously for the whole protein. Since both, the c-MYC protein as well as the non-homologous peptides used for immunization and restimulation contained both predicted MHC class I and class II epitopes, we next investigated whether the T-cell responses observed can be attributed to the CD4^+^ or the CD8^+^ compartment, or both. In these experiments, we incubated APC with c-MYC or OVA in the presence of a monoclonal, anti-human c-MYC antibody (clone 9E10) to enhance uptake and presentation of exogenous proteins [28]. Intracellular IFNγ staining of splenic T-cells from immunized mice revealed activation of both, CD4^+^ and CD8^+^ T-cells when restimulated with human c-MYC protein in comparison to stimulation with OVA. As shown in Figure 1C, flow cytometric analysis after intracellular IFNγ staining identified 1.55% CD4^+^ and 2.82% of CD8^+^ responding T-cells after restimulation with c-MYC loaded APC. In contrast, OVA-loaded APC neither stimulated CD4^+^ nor CD8^+^ T-cells and there was no difference in IFNγ secretion when compared to isotype control staining. 

**Table 1:** Epitope prediction for non-homologous peptides in the context of H2^b^.

**NHP**	**Sequence**	**MHC**	**Core sequence**	**Differences Human - > Mouse**	**Affinity Peptide-MHC**
A	TMPLNVSFTNRNYDLDYDSVQPYFYCDEEENFYQQQQQSEL	H2-Db H2-Kb H2-IAb	VSFTNRNYDL VSFTNRNYDL LNVSFTNRN	S- >N S- >N S- >N	410 nM 82 nM 408 nM 40aa
B	DSSSPKSCASQDSSAFSPSSDSLLSS(T)ESSPQGSPEPLVL	H2-Db H2-Kb H2-IAb	SSPQGSPEPL SAFSPSSDSLL FSPSSDSLL	Q- >R, G- >A S- >T	248 nM 176 nM 458 nM 30aa
C	VEKRQAPGKRSESGSPSAGGHSKP	H2-IAb	SESGSPSAG	P- >S, S- >P, A- >S, G- >A	-1,4 µM 24aa
D	TAYILSVQAEEQKLISEEDLLRKRREQL	H2-IAb	YILSVQAEE	V- >I, E- >D	-7 µM 15aa
E	PSYVAV()TPFSLRGDNDGGGGSFSTADQLEMVTELLG	H2-Kb H2-IAb	VAV()TPFSL YVAV()TPFSL	- >A, P- >S, L- >P - >A, P- >S, L- >P	322 nM 224 nM 36aa
F	PAAKRVKLDSVRVLRQISNNRKCTSPRSSDTEENVKRRTHNV	H2-IAb	RKCTSPRSS	T- >S	-2,7 µM 40aa
G	YQAARKDSGSPNPARGHSVCS	H2-IAb	KDSGSPNPA	G- >T, P- >L, N- >S	-1,2 µM 21aa
H	LYLQDLSAAASEC	H2-IAb	YLQDLSAAA	S- >T	-1,1 µM 13aa

Peptides overlapping non-homologous regions (also see Figure S1) of human c-MYC protein were analyzed using the HLA restrictor software. MHC class I (H2D^b^, H2K^b^)-restricted epitopes were selected according to their affinity <500 nM and at least one amino acid difference. Affinity >50nm is considered weak binding. MHC class II (H2-IA^b^) epitopes were chosen according to differences in amino acid sequence and best binding affinity depending on peptide length, since MHC class II epitopes can vary in length. While length of MHC I epitopes is considered fixed, length (aa) of chosen MHC II epitopes is given next to peptide-MHC affinity and only the core sequence is shown.

### Mapping of immunogenic regions by single peptide immunization

**Figure 2:**
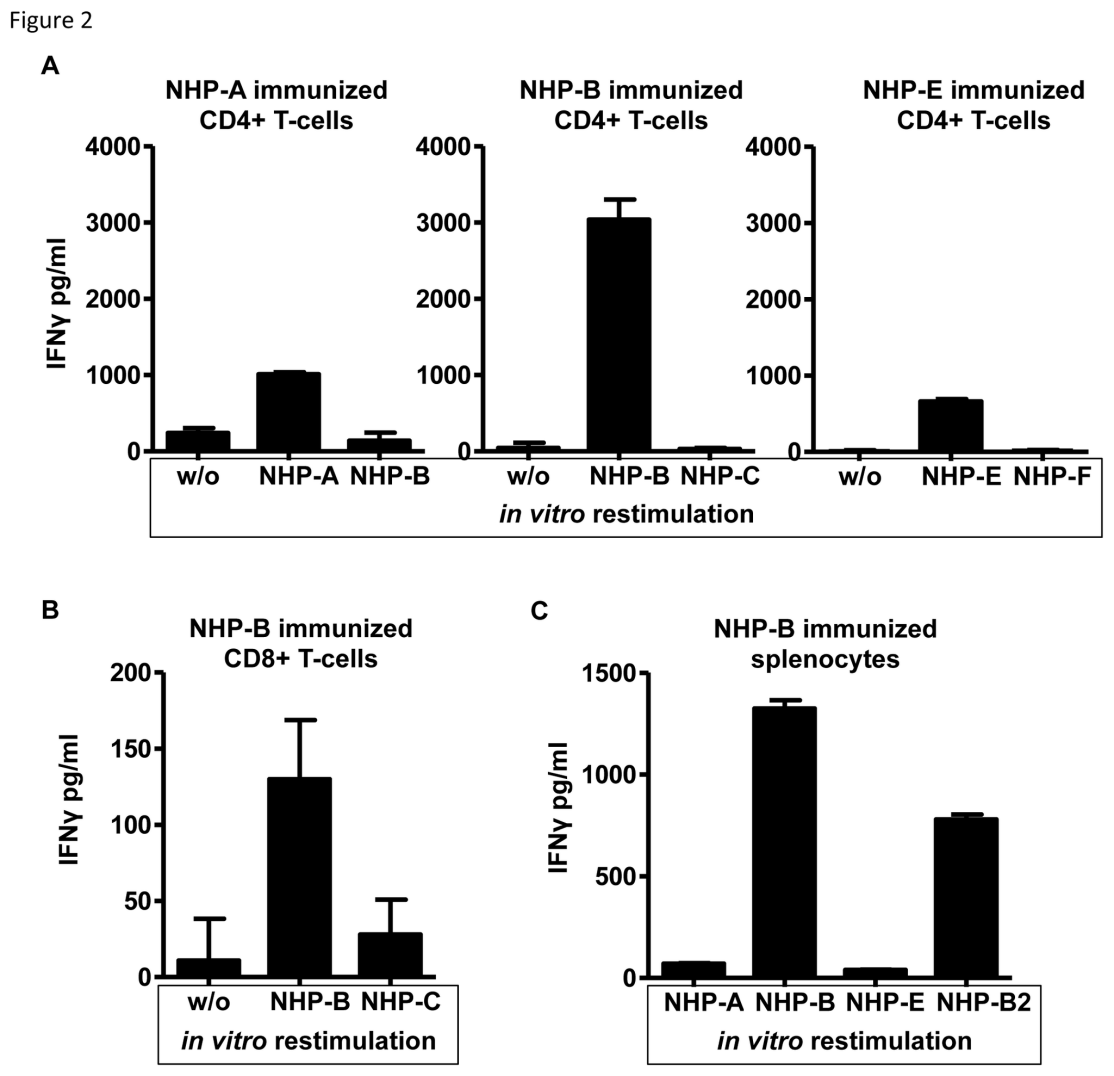
Non-homologous peptides induce CD4^+^ and CD8^+^ T-cell responses. A: CD4+ T-cells were isolated from spleens of mice immunized with single peptides as indicated. T-cells were restimulated for 48h in the presence of unloaded control APC (w/o) or APC loaded with the respective NHP used for immunization. Peptides not used for immunization served as controls. IFNγ secretion was measured by ELISA of culture supernatant. Representative results from three independent experiments are shown. B: CD8^+^ T-cells were restimulated in a similar fashion. Only NHP-B immunization/restimulation resulted in IFNγ secretion. C: Splenocytes from NHP-B immunized mice were restimulated with long peptides and a predicted short H2D^b^ epitope (NHP-B2, 10mer, SSPQGSPEPL) derived from NHP-B. IFNγ secretion was measured by ELISA of culture supernatants (representative example of one out of three independent experiments using 3 animals per group).

**Figure 3:**
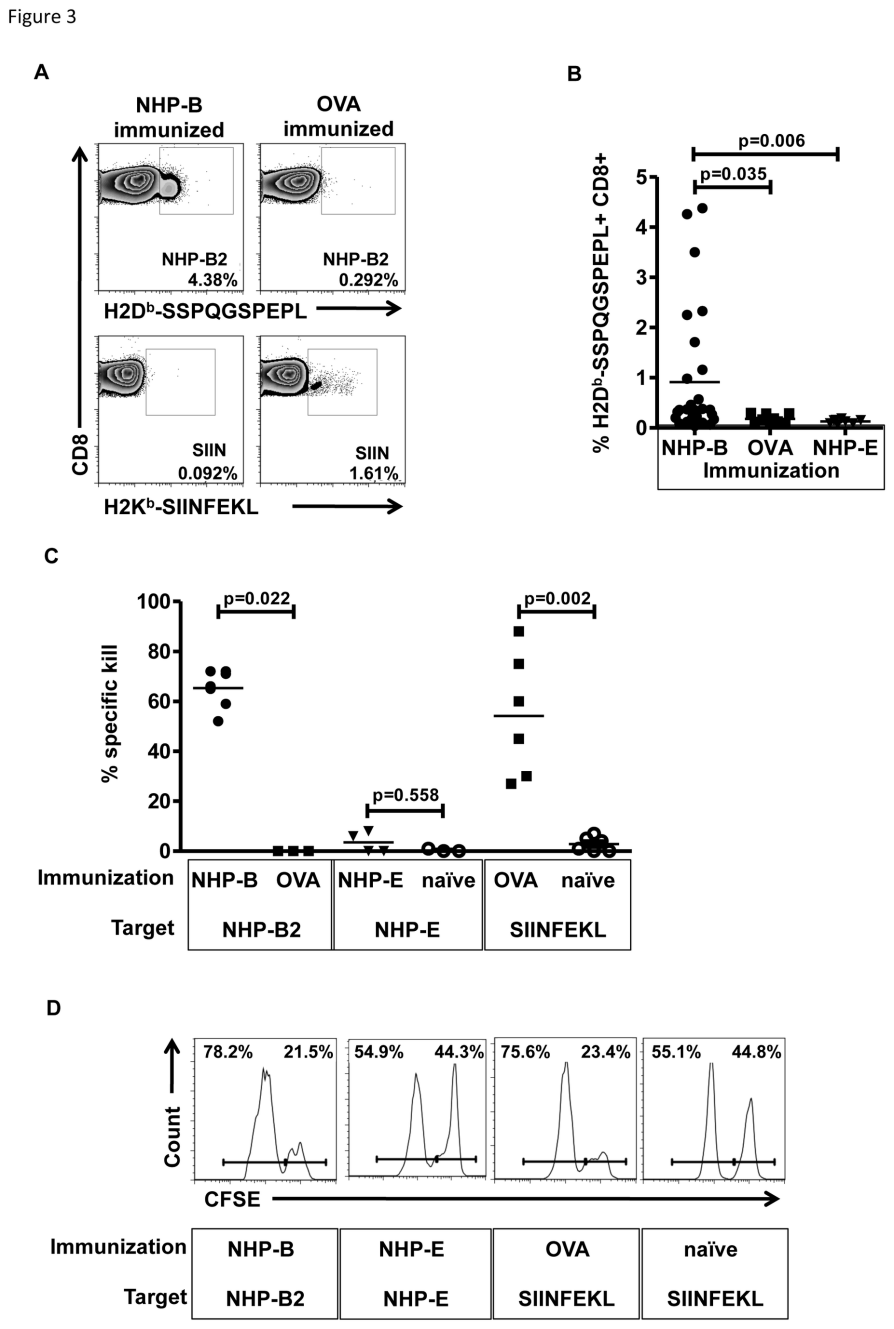
NHP-B immunization induces SSPQGSPEPL-specific CD8^+^ T-cells with cytolytic activity. A: Flow cytometric analysis of peripheral blood mononuclear cells from immunized mice (NHP-B vs. OVA) 7 days after boost immunization using either H2D^b^ multimer-loaded with NHP-B2, or H2K^b^ multimer loaded with SIINFEKL for control. B: Comparison of the frequencies of NHP-B2-specific CD8^+^ T-cells in individual mice after boost immunization according to different vaccines (NHP-B n=28, OVA n=9, NHP-E n=8). C: *In* *vivo* killing assay: animals were immunized with NHP-B, NHP-E or OVA. Naive animals served as controls. Animals were challenged with peptide-pulsed target splenocytes and percentage of specific killing was assessed 18 hours after injection. D: Representative examples of flow cytometric analysis of *in* *vivo* killing assay.

In order to identify more closely which non-homologous region within the peptides contained immunogenic regions, we performed single peptide immunizations. Mice were immunized twice in a similar fashion using single NHPs (A-H, shown in Table 1 and Figure S1). On day 28 after first immunization, spleens and inguinal lymph nodes from two mice were harvested and pooled, CD4^+^ T-cells were enriched by magnetic bead separation (80-90% purity) and re-stimulated with APC pulsed with the specific NHP used for immunization or an irrelevant control peptide as designated. Tissue culture supernatants were harvested after 48 hours. As shown in Figure 2A, splenic CD4^+^ T-cells from mice immunized with either NHP-A, -B or -E responded to restimulation with the specific peptide. All other peptides (C, D, F, G, and H) did not result in specific release of IFNγ (data not shown). Restimulation of CD8+ T-cells with NHP-B-pulsed APC resulted in low amounts of IFNγ in the supernatant (Figure 2B). All other NHPs failed to induce detectable IFNγ release. To verify the assumption that NHP-B contained both a MHC-class II and an additional MHC class I-restricted epitope, we stimulated whole splenic cells in addition to NHP-B with an H2D^b^ restricted NHP-B-derived 10mer (NHP-B2, SSPQGSPEPL) which resulted in IFNγ release (Figure 2C). These results showed that CD8^+^ T-cells recognized a 10-mer of NHP-B. In fact, the NHP-B2-specific CD8^+^ T-cells could be detected by MHC class I H2D^b^ NHP-B2-loaded multimers after immunization. As shown for one mouse in Figure 3A, up to 4.38% of all CD8^+^ T-cells in the peripheral blood stained multimer positive. In contrast, CD8^+^ T-cells from OVA-immunized control animals failed to bind the SSPGQSPEPL multimer, but displayed binding of SIINFEKL multimer (1.61% of CD8^+^ T-cells). Due to the relatively low binding affinity of NHP-B2 to H2D^b^ (Table 1), we analyzed peripheral blood of individual animals 7 days after boost immunization. As shown in Figure 3B, T-cell responses varied considerably among individual animals. The mean percentage of NHP-B2 multimer-binding CD8^+^ T-cells was, however, significantly higher compared to control mice. To test whether the presence of NHP-B2 multimer-binding CD8^+^ T-cells correlated with functional activity, we performed an *in vivo* killing assay. As shown in Figure 3C, NHP-B-immunized animals displayed specific killing of NHP-B2 peptide-pulsed splenic target cells, whereas OVA-immunized animals did not (p=0.022). In contrast, NHP-E-immunized animals did not show any specific lysis of NHP-E-pulsed targets in comparison to naïve mice (p=0.558). Of note, the amount of lytic activity observed for NHP-B-immunized animals was comparable to the positive control, OVA-immunized mice receiving SIINFEKL-pulsed targets as shown in Figure 3D.

### Immunization with NHP-B protects against lymphoma challenge

**Figure 4:**
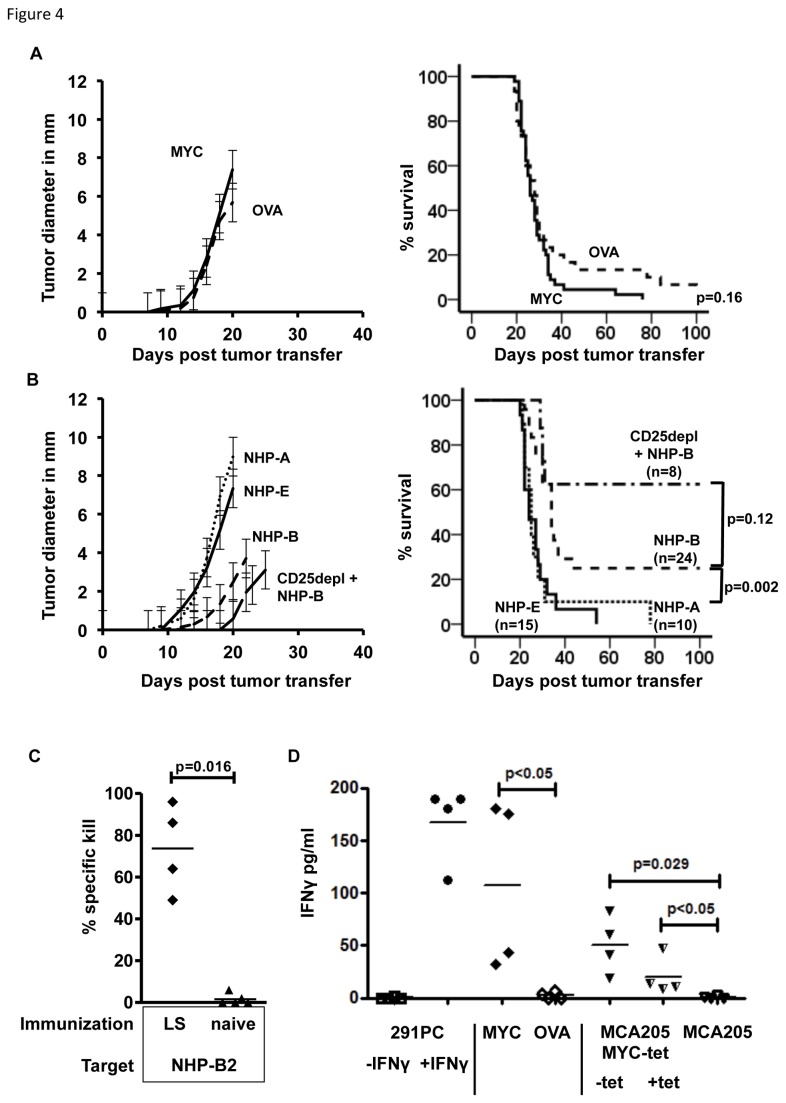
Immunization and challenge with c-MYC over-expressing lymphomas. Immunized recipient mice were challenged with human c-MYC over-expressing B-cell lymphoma cells subcutaneously 7 days past boost immunization. When tumors reached a diameter of more than 10 mm, mice were sacrificed and survival was plotted. Combined data from 3 independent experiments are shown. A: Mice were immunized with a vaccine containing c-MYC protein (solid line, n=45) or OVA protein (dashed line, n=30). Lymphomas grew out simultaneously in both groups and no significant difference in survival was observed (p=0.16). B: Vaccine comprised NHP-A (dotted line, n=10), NHP-E (solid line, n=15) or NHP-B (dashed line, n=24). NHP-B immunization resulted in delayed lymphoma outgrowth and significantly improved survival compared to NHP-A (p=0.006) and NHP-E (p=0.001) vaccination. Depletion of CD25^+^ cells prior to immunization with NHP-B further delayed lymphoma outgrowth and improved survival (CD25depl+NHP-B). C: *In* *vivo* killing assay: lymphoma surviving animals (LS) were challenged with NHP-B2-pulsed splenocytes and percentage of specific killing was assessed 18 hours after injection. Lymphoma surviving animals display a significantly higher *in* *vivo* killing activity compared to naïve animals (p=0.016, Mann-Whitney test). D: IFNγ secretion after stimulation of splenocytes from lymphoma-rejecting mice using c-MYC over-expressing 291PC stimulator cells with (+IFNγ) or without (-IFNγ) prior IFNγ treatment (100U/ml), c-MYC or OVA protein, MCA205MYC-tet with (+tet) and without (-tet) prior tetracycline treatment (1µg/ml) or untransfected MCA205 cells (C and D, n=4 animals per group).

Next we asked whether the lytic activity against splenic targets would also result in an anti-tumor response after immunization with c-MYC protein or single NHPs. Therefore, we challenged immunized mice with 291PC lymphoma cells derived from λ-hu-c-myc transgenic mice. This B-cell lymphoma line expresses the human c-MYC protein in a B-cell specific fashion [20]. As shown previously [25], when 1x10^5^ 291PC cells were subcutaneously inoculated into naïve C57BL/6 mice, animals died of local and - importantly - systemically progressive lymphoma. This demonstrated that the non-homologous regions of xenogenic c-MYC protein, when expressed by malignant B-cells, were not sufficient to induce a protective T-cell response. As shown in Figure 4A, when animals were immunized with c-MYC protein prior to lymphoma challenge, tumors grew out at the site of injection at the same pace as in OVA-immunized control animals (Figure 4A left panel). Lymphoma growth resulted in 90-100% mortality in both groups (Figure 4A right panel, p=0.16). However, when single peptides were used for vaccination prior to lymphoma challenge, we observed a partial protection from lymphoma growth for NHP-B. Lymphoma growth was delayed in these animals (Figure 4B left panel, dashed line) and approximately 25% of animals rejected lymphoma cells long term (Figure 4B right panel). The survival rate after vaccination with NHP-B could be further increased to up to 60%, if animals were treated prior to immunization with an anti-CD25 antibody that is known to deplete CD25^high^ cells, including T-regulatory cells (Figure 4B, CD25depl+NHP-B). In contrast to immunization with NHP-B, single peptide immunization using NHP-A or –E had no protective effect on lymphoma growth and mortality. 

### Lymphoma survivors display T-cell reactivity *in vivo* and *in vitro*

To analyze if rejection of lymphoma cells was associated with immunological memory against NHP-B presenting target cells, we challenged lymphoma surviving animals with peptide-loaded splenocytes and assessed *in vivo* killing activity. Animals surviving lymphoma challenge for more than 100 days were boosted with NHP-B and NHP-B2-pulsed target cells were injected i.v. 7 days after boost. Similarly to the results shown in Figure 3C, lymphoma surviving (LS) animals displayed 50-90% specific killing activity, whereas naïve control mice did not (Figure 4C). In addition, splenic cells from LS animals were restimulated *in vitro* using different targets cells. In these animals, the frequency of SSPQGSPEPL multimer-binding splenic CD8^+^ T-cells was low and ranged from 0.3% to 0.9% at day 100 after lymphoma challenge (data not shown). As shown in Figure 4D, when 2.5x10^5^ spleen cells were incubated with 1x10^4^ 291PC lymphoma cells (E:T ratio 0.08-0.23:1 with respect to multimer-binding CD8^+^ T-cells), we did not observe any secretion of IFNγ although these cells express human c-MYC. However, when 291PC cells were incubated with 100U/ml IFNγ for 24h prior to co-incubation and IFNγ was washed out before adding splenocytes, we observed a significant increase in IFNγ secretion. As shown previously, treatment of 291PC cells with IFNγ increases MHC I and II [25] and leaves c-myc RNA levels upregulated (Figure S3 left panel). IFNγ secretion could also be detected when splenocytes were stimulated with c-MYC protein in presence of anti c-MYC monoclonal antibody (9E10) but not if OVA protein was added instead of c-MYC. In addition, restimulation of splenocytes from lymphoma surviving mice with a murine fibrosarcoma cell line, stably transfected to express human c-MYC (MCA205MYC-tet), also resulted in a significantly higher IFNγ secretion compared to untransfected MCA205 cells. c-MYC expression in MCA205MYC-tet cells can be negatively regulated by tetracycline (tet-off, Figure S3 right panel). Interestingly, IFNγ decreased when MCA205MYC-tet cells were pretreated with 1.0 µg/ml tetracycline 24h prior to coculture with splenocytes. This pointed towards a dose dependent recognition of target cells by NHP-B specific T-cells. Untransfected MCA205 cells did not induce IFNγ secretion by splenocytes from LS mice. 

### Survival after lymphoma challenge is associated with the presence of SSPQGSPEPL-recognizing T-cells

**Figure 5:**
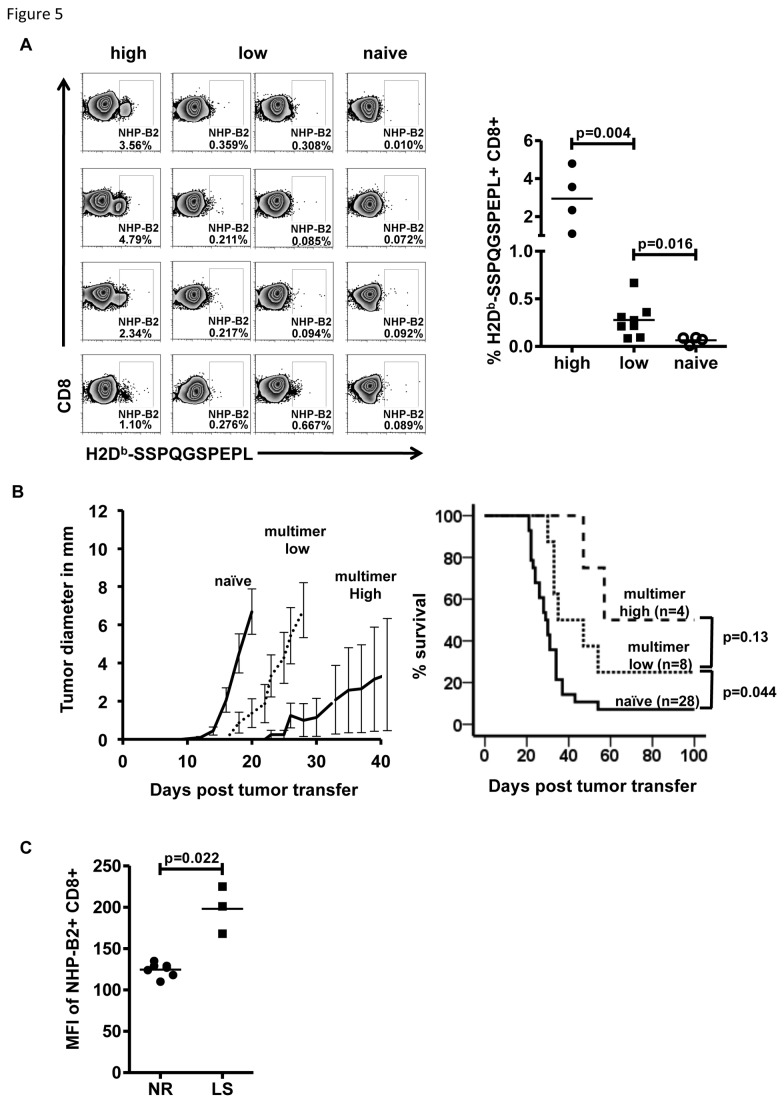
Affinity and frequency of multimer-binding T-cells determine survival after lymphoma challenge. A: Frequency of NHP-B2-specific peripheral blood CD8+ T-cells determined by flow cytometric multimer staining. Animals were segregated into two groups: multimer high >1% of CD8^+^ T-cells, multimer low <1% of CD8^+^ T-cells. Each graph represents one animal. For comparison, multimer staining of 4 naïve animals is shown. The right panel displays the statistical analysis of animals analyzed. B: Survival curve and tumor growth according to multimer grouping. C: Mean fluorescence intensity (MFI) of peripheral blood multimer-binding CD8^+^ T-cells as indicator for T-cell avidity. Lymphoma-surviving animals (LS) display higher avidity compared to not rejecting (NR) recipients prior to lymphoma challenge.

Since immunization with NHP-B led to the induction of a CD8^+^ T-cell response and improved survival after lymphoma challenge, we analyzed if the frequency of NHP-B2-specific, SSPQGSPEPL-multimer-binding T-cells correlated with improved long-term survival. As shown in Figure 5A for individual animals, immunization and multiple boosts with NHP-B induced multimer-binding CD8^+^ T-cells in the peripheral blood with varying frequencies (also see Figure 3B) that can be segregated into two groups: above 1% multimer-binding CD8^+^ T-cells (high) and below 1% multimer-binding CD8+ T-cells (low). The right panel of Figure 5A illustrates the distribution of NHP-B2 multimer-binding CD8^+^ T-cells, which was significantly different between the two groups and from naïve animals. When growth of lymphomas and survival was analyzed according to grouping of animals into multimer-high and -low groups (Figure 5B), we observed a delay in lymphoma growth compared to naïve animals and a significant difference in survival (p=0.044, multimer-low vs. naïve). Multimer-high animals displayed a delay in tumor outgrowth with a tendency to prolonged survival, but due to low numbers of mice in both groups, this did not reach statistical significance (p=0.13, multimer-high vs. multimer-low). However, when we analyzed binding of NHP-B2 multimer in animals that failed to reject 291PC cells (NR) compared to lymphoma survivors (LS), we observed a significant difference in multimer-binding capacity of CD8^+^ T-cells (p=0.022, Figure 5C). 

## Discussion

The work presented here aims to lay the groundwork for c-MYC-specific adoptive T-cell therapy. The use of adoptively transferred T-cells to fight cancer has been effective in a variety of animal models [31], and also in first clinical applications [32,33]. The therapeutic efficacy of these T-cell-based approaches is dependent on several factors. On one hand, there are important prerequisites on the side of the T-cells. For example, the proper functionality of T-cells, which is often associated with IFNγ secretion, is of importance. Similarly, avidity of the T-cell for the target antigen is critical and many strategies were suggested to identify and obtain high affinity T-cells or TCRs to improve adoptive T-cell transfer [34-36]. On the other hand, there are a number of factors on the side of the tumor cell itself and the tumor host, that critically influence effectiveness of adoptively transferred T-cells. For example, general immunogenicity of the tumor target, the immunosuppressive microenvironment, age of the host, and pretreatment may influence anti-tumor therapy (ATT). One of the most critical variables is the tumor antigen. Here, several aspects need to be taken into account: (i) The targeted antigen has to be expressed within the tumor at amounts that allow for recognition in the context of MHC. (ii) The antigen processing and presentation machinery needs to be functional. (iii) The antigen should ideally be expressed in a tumor-specific fashion and not by other tissues, to avoid autoimmunity. (iv) Furthermore, to reduce the risk of antigen loss variants that may cause relapse, the dependency of the malignancy on expression of the antigen is important. Many models have demonstrated that high avidity T-cells will select for antigen loss variants within the tumor, if the malignant phenotype and growth of the tumor remain unaffected by shutting down antigen expression [37-39]. We have previously shown that antigen loss variants occur in the same model used for this study, when chicken ovalbumin (OVA) as a model antigen is targeted by specific OT-1 T-cells [25]. In this model, outgrowth of lymphomas expressing OVA is delayed and only OVA-negative lymphomas arise in immunocompetent hosts. When untransduced 291PC cells were injected into wild type C57BL/6 animals, we also observed a delay in growth compared to STAT-1^-/-^ recipients, which harbor a severe T-cell defect. We suspected from the delayed outgrowth in T-cell competent mice that the expression of human c-MYC might render 291PC cells antigenic, suggesting that the human c-MYC protein might represent a potential T-cell target. Since various oncogene activation pathways converge on c-MYC and malignant growth has been shown to be dependent on c-MYC over-expression, T-cell-based approaches against c-MYC may be effective against different types of tumors and are unlikely to result in the development of antigen loss variants.

In this study, we aimed at identifying c-MYC-reactive T-cells to target a poorly immunogenic c-MYC-overexpressing B-cell lymphoma. In contrast to other studies that successfully used ATT to reject solid tumors of clinically relevant size [31] [40] and often utilize highly immunogenic tumors, i.e. regressor tumors, the 291PC lymphoma target cells do not display a regressor phenotype [25] [40]. To our surprise, we did not observe any difference in lymphoma growth after immunization with full-length c-MYC protein and subsequent challenge with 291 PC cells, even though we observed c-MYC-reactive CD4^+^ and CD8^+^ T-cells *ex vivo*. This finding was compatible with two not mutually exclusive possibilities: either the immunization protocol was suboptimal and the induced T-cells were not of high affinity, or regulatory T-cells were induced in parallel that suppressed the potentially protective response. 

As an alternative to whole protein immunization we used peptides for vaccination encompassing the regions of highest amino sequence divergence. Contrary to most peptides that induced only a weak CD4^+^ and no CD8^+^ T-cell response, the 40mer NHP-B peptide, that differs by only five amino acids to the murine sequence (Figure S1), was the best IFNγ inducer for CD4^+^ cells and induced also significant amounts of IFNγ in CD8^+^ T-cells. Yet, contrary to previous experiments in which OVA proved to be a highly effective T-cell rejection antigen [25], the presence of NHP-B reactive T-cells resulted in an only modest delay in tumor outgrowth. This is in accordance with the predicted low binding affinity of the target peptide to mouse MHC-I (predicted 248 nM). 

Another reason for the failure of whole c-MYC protein or low efficacy of the large peptide vaccine to protect mice against lymphoma challenge might be the presence of regulatory T-cells. Depletion of T-regulatory cells has been shown to increase T-cell reactivity towards auto-antigens *in vivo* upon immunization [41]. Highly homologous proteins like human c-MYC with 89.9% amino acid identity to murine c-Myc may be particularly prone to activation of Tregs. In fact, Tregs proved to play an important role in limiting the immune response to human c-MYC, as Treg depletion with anti-CD25 antibody PC61 prior to immunization increased the survival of NHP-B-immunized animals after lymphoma challenge from 25% to 62.5%. 

This experiment provided evidence that the T-cell response induced against NHP-B was, at least in part, protective. It seems that in particular CD8^+^ T-cells with specificity for SSPQGSPEPL had protective potential, since *in vivo* cytotoxic activity correlated with both survival after lymphoma challenge and with increased frequency of multimer-binding cells. Therefore, our study provides first proof of principle that immunologically targeting c-MYC may result in anti-tumor activity. The precise role of CD4^+^ and CD8^+^ T-cells in this system has, however, not yet been fully elucidated. In other mouse tumor systems, CD8^+^ and CD4^+^ T-cells have been shown to confer not only cytotoxic activity (CD8^+^) and help for other T-cells (CD4^+^), they also orchestrate the immune response against tumor cells by activating cells of the innate immune system, i.e. NK cells and macrophages, in an antigen-dependent manner [42,43]. CD4^+^ T-cells furthermore play a crucial role in the induction of cellular senescence and angiogenesis arrest in c-Myc-addicted tumors when *c-Myc* expression is shut off [44]. It remains to be elucidated which cells of the immune system contribute to anti-cMYC-specific anti-tumor activity and whether c-MYC NHP-B specific CD4^+^ T-cells are required for long term tumor rejection in this model system. 

In addition, several aspects remain that need further investigation: the most important concern is autoimmunity due to the expression of c-MYC in healthy, non-malignant proliferating tissues. In normal tissue c-MYC expression is tightly regulated [45], whereas in lymphomas and other tumors c-MYC is invariably deprived of its physiological control and constitutively switched on. The hematopoietic and the gastrointestinal system depend on c-MYC for tissue homeostasis and may be regarded as the most critical tissues for targeted anti-c-MYC therapy. In a murine K-RAS-driven lung cancer model designed to reversibly inhibit c-MYC, c-MYC inhibition not only induced regression of incipient and established lung tumors, it also exerted profound effects on normal regenerating tissues, as might be anticipated. Yet, these effects were well tolerated over extended periods of time of up to 60 days and were completely reversible [11]. This is encouraging and may suggest that side effects of anti-c-MYC-specific T-cells may also be tolerable. To address whether a therapeutic window exists between anti-tumor immunity of c-MYC-specific T-cells and autoimmunity, we have generated a humanized c-MYC mouse in which the endogenous murine *c-Myc* gene is replaced by the human *c-MYC* gene. This mouse expresses the human c-MYC protein under the physiological control of the endogenous murine *c-Myc* promoter, is viable, and does not display an obvious phenotype [46]. This mouse will allow us to investigate whether tissue expression of the antigen causes autoimmunity when c-MYC specific T-cells are administered. Another concern may be that T-cell responses against c-MYC will induce fratricide, as has been observed for survivin [47], because c-Myc is induced upon activation in T-cells [48]. These issues have to be experimentally addressed and need to be clarified in the mouse model before c-MYC-specific T cell therapy can be introduced into the clinic.

The high homology between human c-MYC and murine c-Myc is limiting the pool of divergent peptides to which a strong T-cell response may be elicited in the context of a given MHC class I allele. For anti-c-MYC-specific T-cell therapy in humans, it will be decisive whether a potent T-cell response can also be elicited in HLA-A2- and huTCR-transgenic mice [49] [36]. If so, T-cells of HLA-A2-positive cancer patients may be equipped with HLA-A2/c-MYC-specific TCR genes by retroviral gene transfer and such T-cells administered to HLA-A2-positive cancer patients. 

However, the greatest challenge will be to generate novel genetic mouse models in which a much broader T-cell response against human c-MYC can be elicited. Notably, mice are viable and fertile in which the endogenous c-Myc gene has been replaced by the murine N-Myc gene [50]. The homology between human c-MYC and murine N-Myc protein is only 37% amino acid identity and 52% similarity suggesting that a much broader T-cell response against human c-MYC may be raised in these mice, provided these mice are able to elicit a normal T-cell response. Importantly, the response of lymphocytes to mitogens does not appear to be impaired in these mice suggesting that such an approach may indeed be feasible.

In conclusion, our work has illustrated for the first time the principal feasibility of targeting human c-MYC with T-cells. Although c-MYC-specific adoptive T-cell therapy is still at its infancy, it may represent on a long run a highly attractive and promising novel tool that may complement classical anti-cancer chemotherapy. 

## Supporting Information

Figure S1 **Comparison of murine and human c-myc amino acid sequence.** Non homologous peptides (NHP) used for the study are framed.
(TIF)

Figure S2 **IFNγ secretion after immunization and *invitro* restimulation.** 1x10^5^ T-cells from mice immunized with full-length c-MYC protein and boosted with a NHP pool (MYC-NHP) were cultured for 48 hours alone (w/o) or in the presence of 1x10^4^ APC pulsed with either c-MYC, OVA or NHP pool. T-cells from OVA immunized and naïve mice served as controls.
(TIF)

Figure S3 **Left panel: Human c-myc mRNA is expressed in 291 lymphoma cells and upregulated upon IFNγ treatment.** Human *c-myc* cDNA was analyzed by quantitative real time PCR in murine splenocytes for control (SPL) and 291PC cells treated with or without IFNγ (100 U/ml) for 24 hours before RNA extraction. Right panel: Human c-MYC is expressed in MCA205MYC-tet sarcoma cells. Western blot analysis using human c-MYC specific antibody 9E10 reveals c-MYC protein in MCA205MYC-tet cells. After treatment withincreasing doses of tetracycline (0.1 and 1.0 µg/ml) for 24 hours, expression of c-MYC is downregulated. Untransfected MCA205 cells do not express human c-MYC. 
(TIF)
