# Supplementary figures and images for: Targeting c-MYC with T-Cells
Source: PLoS One. 2013 Oct 10;8(10):e77375. doi: 10.1371/journal.pone.0077375 (PMC3795085; doi:10.1371/journal.pone.0077375)

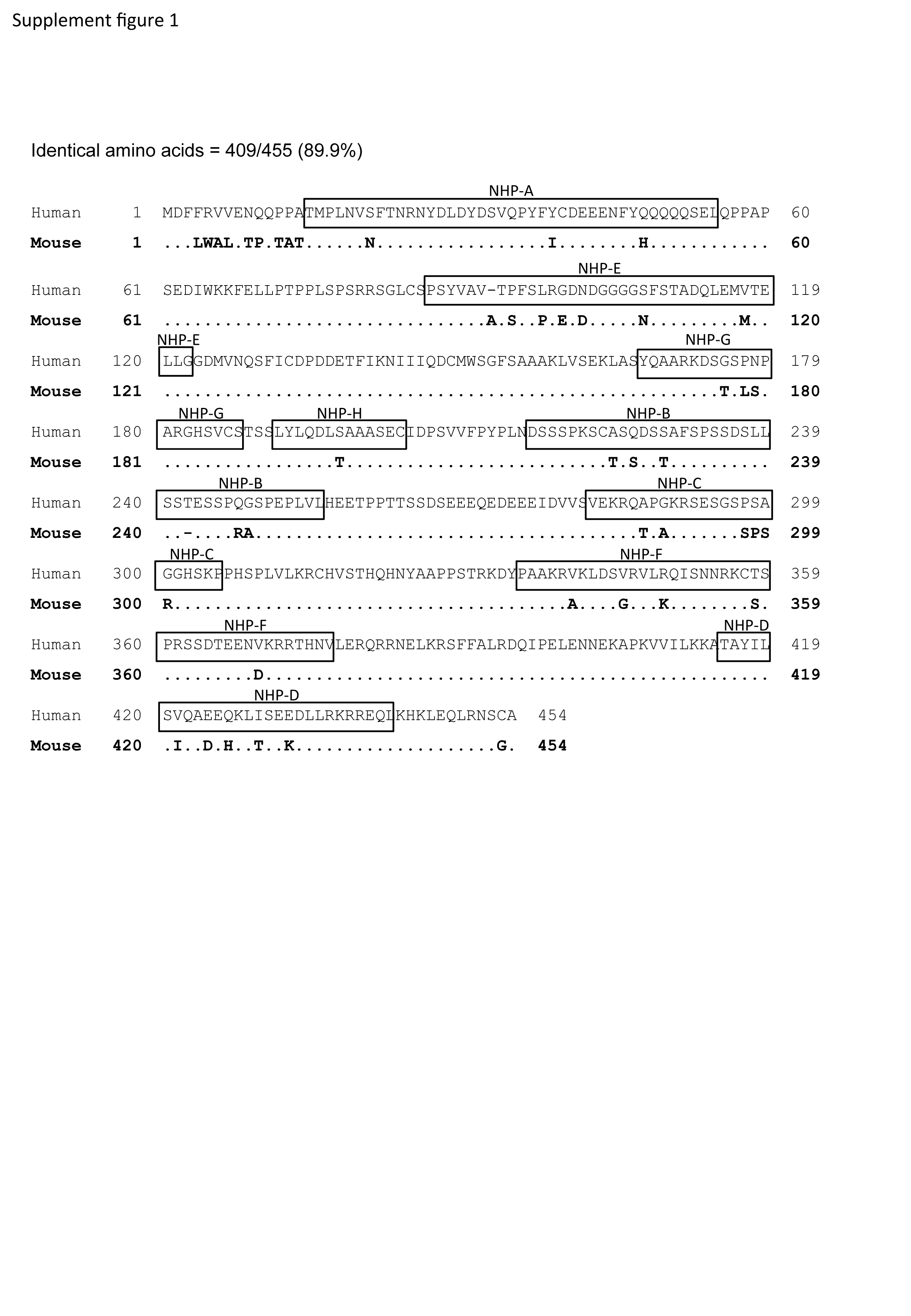

Supplement: Figure S1 — Comparison of murine and human c-myc amino acid sequence. Non homologous peptides (NHP) used for the study are framed. (TIF) [file pone.0077375.s001.tif]

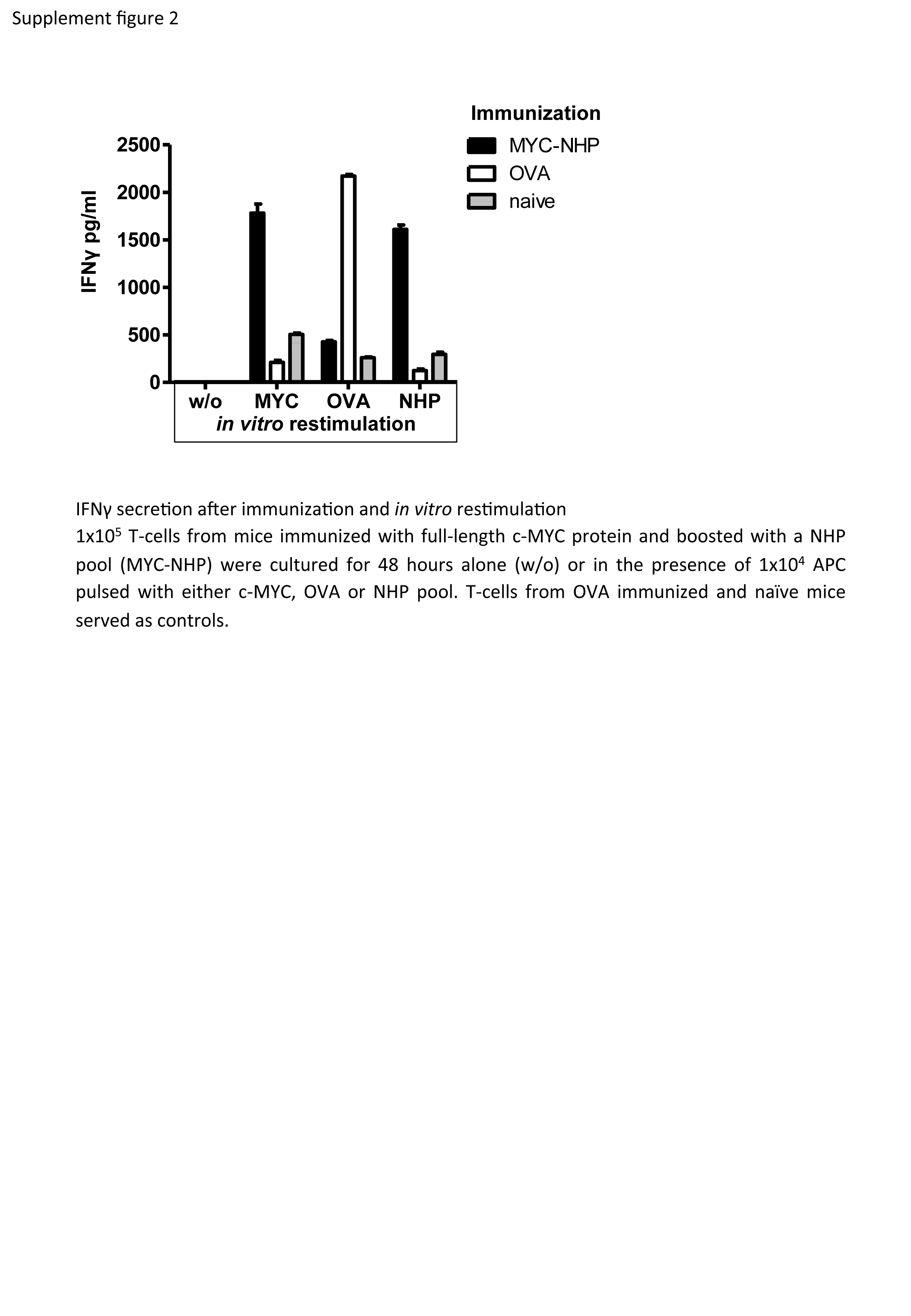

Supplement: Figure S2 — IFNγ secretion after immunization and invitro restimulation. 1x10^5^ T-cells from mice immunized with full-length c-MYC protein and boosted with a NHP pool (MYC-NHP) were cultured for 48 hours alone (w/o) or in the presence of 1x10^4^ APC pulsed with either c-MYC, OVA or NHP pool. T-cells from OVA immunized and naïve mice served as controls. (TIF) [file pone.0077375.s002.tif]

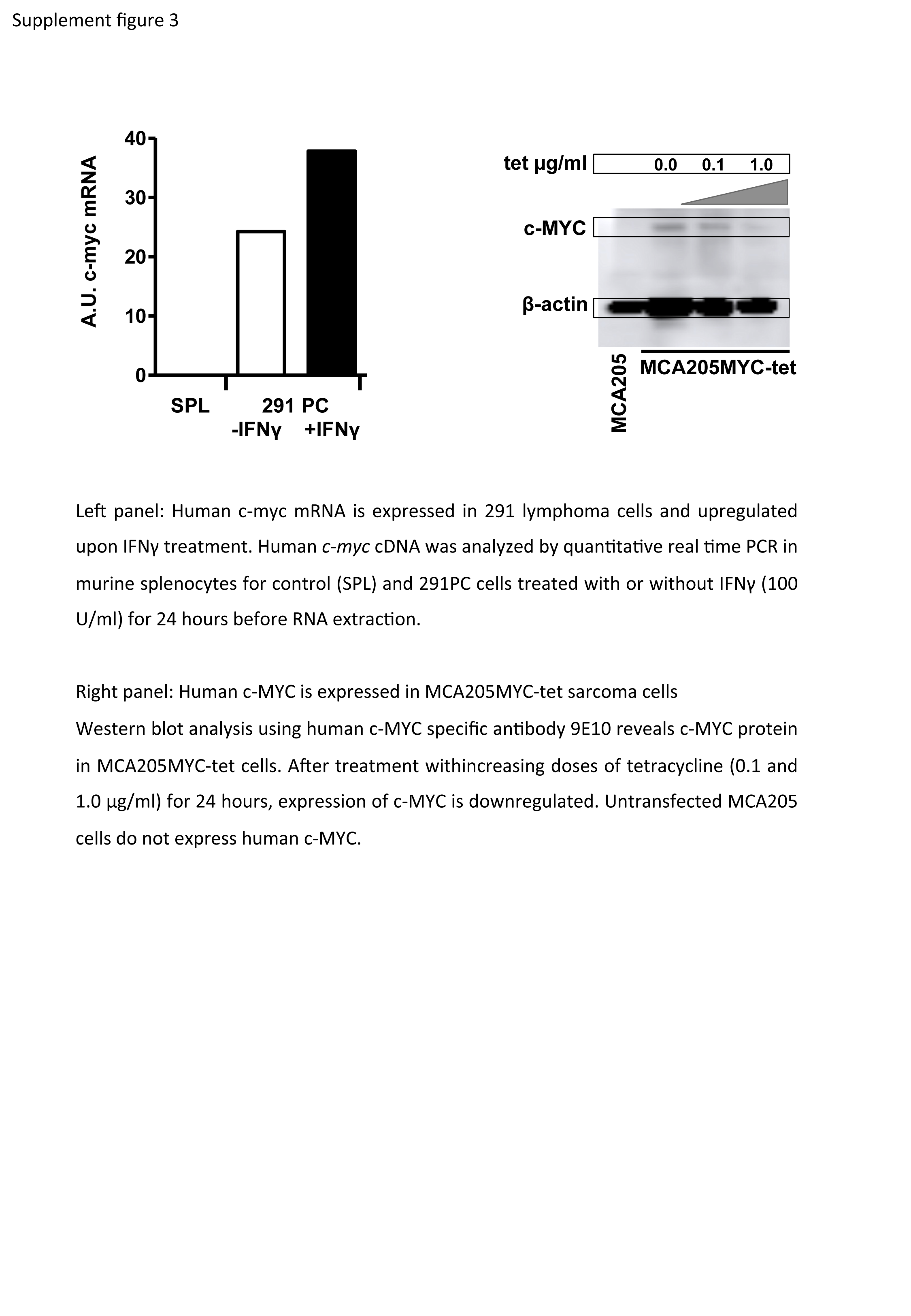

Supplement: Figure S3 — Left panel: Human c-myc mRNA is expressed in 291 lymphoma cells and upregulated upon IFNγ treatment. Human c-myc cDNA was analyzed by quantitative real time PCR in murine splenocytes for control (SPL) and 291PC cells treated with or without IFNγ (100 U/ml) for 24 hours before RNA extraction. Right panel: Human c-MYC is expressed in MCA205MYC-tet sarcoma cells. Western blot analysis using human c-MYC specific antibody 9E10 reveals c-MYC protein in MCA205MYC-tet cells. After treatment withincreasing doses of tetracycline (0.1 and 1.0 µg/ml) for 24 hours, expression of c-MYC is downregulated. Untransfected MCA205 cells do not express human c-MYC. (TIF) [file pone.0077375.s003.tif]
